# Résultats à moyen terme du traitement des ostéochondrites disséquantes des condyles fémoraux par greffe ostéochondrale en mosaïque

**DOI:** 10.11604/pamj.2019.32.191.17308

**Published:** 2019-04-18

**Authors:** Abdallah Elmokhtar, Abderazzak Rafrafi, Talel Znagui, Saber Saadi, Mounira khezami, Mounir Hamdi, Lotfi Nouisri

**Affiliations:** 1 Service d'Orthopédie à Hôpital Militaire Principal de Tunis, Tunis, Tunisie

**Keywords:** Suppurations, paroi, césarienne, CHU/YO, Osteochondritis, femoral condyle, mosaic arthroplasty

## Abstract

Face à une perte de substance cartilagineuse des condyles fémoraux, plusieurs alternatives thérapeutiques interventionnelles sont envisageables y compris la mosaïcoplastie. Le but de notre travail était d'évaluer le résultat clinique et radiologique à moyen terme ainsi que d'évaluer les principaux éléments pronostiques. Notre étude épidémiologique rétrospective étalée sur 15 ans nous a permis de colliger 35 dossiers exploitables d'ostéochondrite disséquante des condyles fémoraux traités par la technique de mosaïcoplastie avec un recul moyen de 24 mois. Le niveau des plaintes ainsi que la fonction du genou en préopératoire ont été étudiés et comparés par rapport au genou sain selon le Score International Cartilage Repair Society (ICRS), le score International Knee Documentation Committee (IKDC) et l'échelle visuelle de la douleur (EVA) et nous avons trouvé qu'elle était inférieure à 60% chez 27 malades. L'évaluation des résultats au recul ont été analysés selon les critères fonctionnels et radiologiques de Hunghston. Après un recul moyen de 24 mois, l'algoneurodystrophie était observée dans 05 cas avec un seul cas d'hémarthrose. Une nette amélioration du score ICRS était observée avec une moyenne qui a passée du 54% à 74% au recul. Les patients satisfaits ou très satisfaits (82,9%) furent largement majoritaires. Les éléments de bon pronostic constatés dans notre étude étaient: un délai opératoire moins de 18 mois de début de la symptomatologie, les lésions profondes ayant un diamètre inférieur à 02 cm et les lésions siégeant au niveau du condyle interne. Le traitement des pertes de substances cartilagineuses passe obligatoirement par la correction des causes directes et indirectes à savoir le morphotype, la laxité et le capital méniscal. Aucun consensus décisionnel n'a pu être proposé et nul ne peut confirmer la supériorité d'une technique par rapport à l'autre mais nous pouvons dire que le défect cartilagineux de 2 à 4 cm^2^ peut être la meilleure indication à la mosaïcoplastie.

## Abstract (en)

Several surgical procedures, including mosaic arthroplasty, can be used to treat patients with cartilage loss in the femoral condyles. This study aims to assess mid-term clinical and radiological results as well as the main prognostics elements. We conducted a retrospective epidemiological study over a period of 15 years. During the study period we collected data from 35 workable medical records of patients with osteochondritis dissecans of the femoral condyles treated by mosaic arthroplasty, with an average follow-up of 24 months. The level of complaints as well as preoperative knee function were evaluated and compared with the healthy knee according to the International Cartilage Repair Society (ICRS) score, the International Knee Documentation Committee (IKDC) score and visual analogue scale (VAS). It was less than 60% in 27 patients. During the follow-up period, the results were analyzed according to Hughston's functional and radiological criteria. After an average follow-up of 24 months, algoneurodystrophy was reported in 5 cases with a single case of haemarthrosis. A net ICRS score improvement was observed with a mean increasing from 54% to 74% on the follow-up visit. Most of patients were satisfied or very satisfied (82.9%). The elements of good prognosis recognized in our study included: a mean time between symptom onset and surgery of less of 18 months, having deep lesions with a diameter less than 02 cm and having lesions in the internal condyle. The treatment of cartilage loss is necessarily based on the correction of its direct and indirect causes namely the morphotype, the laxity and meniscus capital. No consensus in the decision-making was reached and no one could confirm the superiority of a technique in relation to the other but we can say that cartilage defect which sizes from 2 to 4 cm² may be the best indication for mosaic arthroplasty.

## Introduction

Face à une lésion ostéochondrale profonde du genou, le potentiel intrinsèque d'une réparation spontanée est très faiblement expliqué par la pauvreté de la vascularisation cartilagineuse, rendant nécessaire l'intervention chirurgicale pour les réparées. Ce traitement chirurgical reste jusqu'à nos jours un sujet difficile et non codifié. Depuis plus de cinq décennies, plusieurs techniques ont été essayées dans le but de réparer la perte de substance cartilagineuse et d'obtenir un tissu le plus proche possible du cartilage dit « Hyaline-like » à savoir les perforations multiples de l'os sous chondral et la culture de l'injection des chondrocytes autologues [1-4]. La greffe ostéochondrale en mosaïque ou « mosaïcoplastie » est une technique de réparation développée à partir des années 1990 et restent largement utilisée puisqu'elle est plus faciles à mettre en œuvre et moins onéreuse [4]. Elle consiste à prélever des greffons ostéochondraux à partir d'un site donneur bien défini et de les transférer au niveau du défect ostéochondral les uns à côté des autres à la manière d'une mosaïque. Elle présente plusieurs avantages théoriques comme le respect du rayon et de courbure de la surface articulaire ainsi que l'intégration du greffon avec l'os spongieux par l'intermédiaire d'un fibrocartilage qui se forme entre les différentes greffes à partir du sous-sol sous-chondral avivé [3,5]. Nous avons essayé à partir de notre série de 35 militaires actifs présentant une perte de substance cartilagineuse des condyles fémoraux traités par mosaïcoplastie d'évaluer le résultat clinique et radiologique à moyen terme ainsi que d'évaluer les principaux éléments pronostiques.

## Méthodes

**Figure 1:**
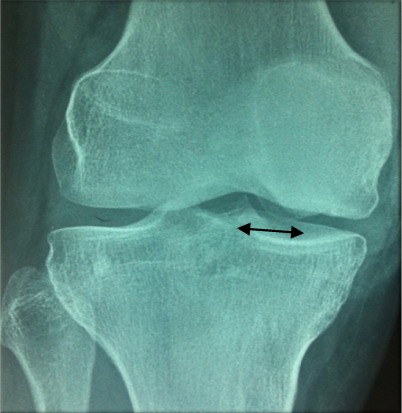
Classification de Cahil et Berg

**Figure 2:**
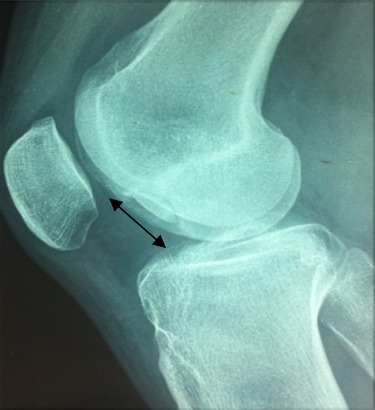
Classification de Harding

**Figure 3:**
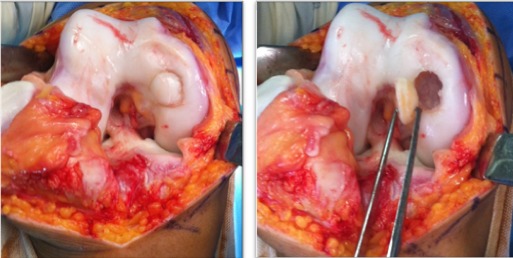
Déroulement de l'intervention

**Figure 4:**
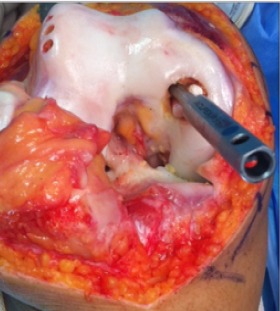
Répartition des genoux selon le score ICRS

**Figure 5:**
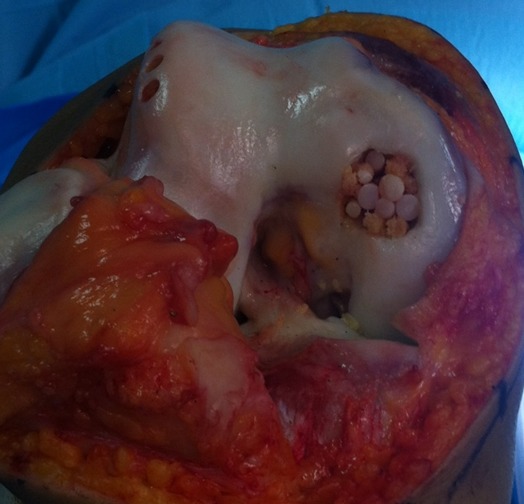
Variation des scores ICRS au recul

Notre étude épidémiologique rétrospective menée au sein du service d'orthopédie et traumatologie de l'hôpital militaire d'instruction de Tunis étalée sur 15 ans entre 2001 et 2015, a porté sur les cas d'ostéochondrite disséquante (OCD) des condyles fémoraux traités par la technique de mosaïcoplastie ayant un recul minimum de 24 mois tout en excluant les malades âgés de moins de 15 ans ou plus de 50 ans. Les malades ayant des lésions d'arthrose au bilan radiologique et les autres localisations d'OCD. Notre étude nous a permis de colliger 35 dossiers exploitables des agents militaires actifs avec une moyenne d'âge de 33,6 ans et une nette prédominance masculine (31 hommes et quatre femmes) avec un recul moyen de 42 mois. Il a été précisé pour chaque malade les données épidémiologique et anthropologique ainsi que le niveau sportif. Notre population était en majorité sportive (seulement une femme était sédentaire). Aucune atteinte bilatérale n'a été notée et trois genoux seulement ont subi une intervention chirurgicale antérieure et qui ont été des cures méniscales par voie arthroscopique. L'évaluation de la fonction du coté lésé ainsi que le niveau des plaintes ont été étudiés selon le score International Cartilage Repair Society (ICRS) [6], le score International Knee Documentation Committee (IKDC) [7] et l'échelle visuelle de la douleur (EVA). Les caractéristiques radiologiques de la lésion en préopératoire ont été étudiées. La localisation a été précisée sur l'incidence de face selon la classification de Cahil et Berg et sur l'incidence de profil selon la classification de Harding (Figure 1, Figure 2). L'étendue de la lésion a été quantifiée par la mesure de sa surface et de son volume. Les stades évolutifs ont été précisés soit par la classification de Bedouelle sur une radiographie standard ou par la classification de Bernet et Hardy pour les genoux explorés par une imagerie par la résonance magnétique [8]. L'intervention se déroule sous anesthésie générale ou locorégionale avec un garrot pneumatique à la racine du membre. Tous les malades ont eu une chirurgie à ciel ouvert par un abord para-patellaire interne (Figure 3). Les greffons ont été prélevés à partir des berges internes et externes de la trochlée avec un nombre moyen de six greffons par genou et une couverture moyenne de 82% de la perte de substance cartilagineuse (Figure 4, Figure 5). Une immobilisation articulaire post opératoire de six semaines, une rééducation active et passive et une proscription d'appui de deux mois ont été appliqués chez tous les malades. En post opératoire, tous les malades ont eu au dernier recul une évaluation clinique globale portant sur des éléments fonctionnels, des données de l'examen physique et la recherche des éventuelles complications évolutives. Aussi, tous les malades ont été explorés par une radiographie standard de genou de face et de profil au dernier recul. Ensuite, les résultats ont été analysés selon les critères fonctionnels et radiologiques de Hunghston [9] établies à partir du score ICRS fonctionnel, des données de l'examen physique et des données de la radiologie standard.

## Résultats

Dans notre population d'étude, la douleur était le maitre symptôme et le motif de consultation dans tous les cas chez près de 20 malades était une cotation EVA inférieure à six. Les autres manifestations telles que le blocage et l'accrochage n'ont pas été observés que dans sept cas. La mobilité articulaire était conservée chez tous les malades et la lésion siégeait au niveau des condyles internes dans 24 cas et était classée III ou IV selon la classification de Bedouelle dans tous les cas. L'évaluation de la fonction du genou atteint par rapport au genou sain selon le score d'ICRS trouve qu'elle était inférieure à 60% dans 27 cas. Après un recul moyen de 24 mois, aucune complication per opératoire n'a été notée, un malade a développé une hémarthrose qui a bien évoluée par la cryothérapie et la thérapie anti-inflammatoire, un cas de thrombophlébite superficielle bien jugulée par un traitement anticoagulant, cinq cas d'algo-neuro-dystrophie qui ont bien évolué sous traitement et deux cas de raideur articulaire mais avec une récupération quasi-complète après un programme de rééducation bien conduit.

Aussi, nous avons observé une nette amélioration du score ICRS des genoux opérés au recul avec une moyenne qui a passée du 54% en préopératoire à 74% au recul et une majorité des genoux classés stade II (Tableau 1). Concernant le résultat clinique global et selon la classification de Hughston, nous avons obtenu six excellents résultats, 23 bons résultats soit un résultat satisfaisant dans 82,9% des cas avec un seul mauvais résultat. Sur le plan radiologique et selon la classification de Hughston, nous avons obtenu 21 excellents résultats, huit bons résultats avec un seul mauvais résultat. D'un autre coté et en analysant nos résultats en fonction des différents paramètres, nous avons remarqué que les genoux opérés après un délai de 18 mois du début de la symptomatologie ont un résultat fonctionnel et des scores ICRS moins bons comparé aux genoux opérés plus précocement mais sans avoir une valeur statiquement significative (p= 0,062). L'âge au moment de l'intervention n'a pas influencé le résultat global dans notre étude et l'analyse des résultats en fonction du sexe n'a pas été possible vu que notre population d'étude était presque totalement de sexe masculin. Aussi, la profondeur, l'étendue et la localisation des lésions ont influencé le résultat global au recul. En effet, les lésions profondes (Stade IV de Bedouelle) ont des résultats meilleurs mais sans significativité statistique (p=0,059), les lésions du condyle interne ont aussi un résultat meilleur mais avec une significativité statistique (p=0,008) et les petites lésions dont le diamètre est inférieure à deux cm étaient des meilleurs scores de Hughston comparé aux lésions plus volumineuses (p=0,03). Enfin, l'obésité, le tabagisme, la notion du traumatisme, le nombre des greffons et le pourcentage de couverture n'ont pas influencé le résultat global au recul.

**Tableau 1:** Variation des scores ICRS au recul

	Score ICRS en pré-opératoire	Score ICRS au recul
**Stade I**	02 cas	07 cas
**Stade II**	06 cas	22 cas
**Stade III**	21 cas	05 cas
**Stade IV**	06 cas	01 cas

## Discussion

La réparation chirurgicale d'une perte de substance cartilagineuse du genou dans le cadre d'ostéochondrite disséquante symptomatique des douleurs invalidantes ou d'un gène fonctionnel important est une décision difficile à prendre en raison de sa complexité ainsi que de ses risques. Elle ne doit pas être envisagée qu'à la demande du malade qui doit être impliqué dans la décision thérapeutique [4]. Une fois l'indication chirurgicale est prise, la greffe cartilagineuse en mosaïque est une alternative thérapeutique de restauration efficace et validée par plusieurs auteurs [10]. Elle a l'avantage d'apporter des greffons ostéochondrales autologues qui ne nécessitent pas des préparations au préalable dans des laboratoires de thérapie cellulaire avec possibilité d'adapter les dimensions des greffons en fonction de la perte de substance. C'est une technique moins onéreuse que les techniques de reconstruction, réalisée en un seul temps opératoire avec des risques septiques et de rejet presque nuls [3,4]. Néanmoins, c'est une chirurgie qui n'est pas dépourvu de risque ou d'inconvénient. Il s'agit d'une technique exigeante qui demande une grande rigueur pour le prélèvement et l'implantation et dépend de l'expérience du chirurgien [3,4]. Hangody *et al.* rapportaient dans une série rétrospective hétérogène de plus de 1000 greffes en mosaïque: 3% de morbidité, quatre infections et 36 hémarthroses. Ce taux a été de 13% dans la série de Ollat D *et al.* avec également une prépondérance des hémarthroses [2,3]. Dans notre série, un seul cas d'hémarthrose a été observé soit un pourcentage de 2,8% et la morbidité était essentiellement dû aux cas d'algo-neuro-dystrophie. Les greffons cartilagineux prélevés et implantés dans la perte de substance vont subir une intégration de la partie osseuse spongieuse de la greffe qui fusionne avec le lit spongieux receveur et une intégration du cartilage transplanté avec le cartilage hyalin par l'intermédiaire d'un fibrocartilage [3]. Ces greffons ont une double stabilité verticale et horizontale. La stabilité verticale est influencée par les dimensions du greffon et la longueur des puits receveurs et la stabilité horizontale dépend de la présence ou non d'un effet press-fit [4]. Duchow *et al.* dans leur étude sur des genoux porcins et en essayant différents diamètres et différents longueurs ont constaté que les greffons de 10 à 11 mm de diamètre et de 15 à 20 mm de longueur ont une stabilité supérieure [11]. Aussi, Kock *et al.* dans une étude cadavérique ont montré que les greffons ajustés à la longueur des puits vont subir moins des forces de pressions axiales et donc plus de stabilité verticale [12]. Makino *et al.*, Pearce *et al.* ont montré que l'association de l'effet press-fit va stabiliser le cartilage qui ne va pas subir une hypertrophie et qu'un léger surdimensionnement en largeur est souhaité pour conserver le caractère mature du cartilage [13,14].

La zone donneuse, laissée vide par la plupart des chirurgiens, va subir des remaniements fibreux laissant une surface en dépression comme montrait l'étude de Ahmad *et al.* basée sur des contrôles arthroscopiques secondaires post opératoires [15]. Cette zone donneuse était le sujet d'un nombre important des travaux scientifiques dans le but de préciser le site optimal, sa morbidité, ainsi que les éventuelles tentatives de comblement. Les berges supéro-médiales de la trochlée étaient le site optimal choisi par Garrseton *et al.* ainsi que Ollat *et al.* puisque elles étaient les zones les moins contraintes [3,16]. Ce site fut celui choisi dans 61% des cas dans notre série. Aussi, le respect de l'épaisseur et de la courbure des greffons est important pour maintenir une bonne répartition des contraintes [17,18]. La morbidité de cette zone donneuse n'est pas nulle et son taux varie dans la littérature entre zéro et 36%. Ces valeurs extrêmes étaient présentées par l'étude de Iwasaki *et al.* [19] qui n'ont rapporté aucune complication des prélèvements chondraux à partir des genoux sains pour des lésions humérales à deux ans de recul contrairement à l'étude de Reddy *et al.* qui ont montré que quatre genoux parmi 11 ont présentés un mauvais résultat à quatre ans de recul [20]. Ollat D *et al.* n'ont trouvé aucune corrélation entre l'importance du prélèvement et la survenue de l'arthrose fémoro-patellaire après un recul de huit ans [3]. Dans le but de diminuer le risque de cette morbidité, certains auteurs ont essayé de combler la zone donneuse par un plot ostéopériosté sans réussir à avoir un comblement osseux de qualité [21]. Les résultats cliniques globaux de la mosaïcoplastie sont habituellement satisfaisants quoique plusieurs scores d'évaluation aient été utilisés par les autres (ICRS, Score de Hughston, Score de Lysholm, Cincinnati Knee Score…). Quel que soit le score d'évaluation utilisé, la satisfaction du malade de résultat était obtenue dans toutes les séries avec des taux qui varient entre 74% et 92%. Le Tableau 2 montre les résultats fonctionnels des principales séries étudiées. Des auteurs ont publié leurs résultats pour des séries qui s'intéressaient seulement aux lésions condyliennes et ont montré des résultats satisfaisants avec des taux qui varie entre 84% et 88% [22,23]. Tous ces auteurs insistent sur la supériorité de la mosaïcoplastie en termes de simplicité, apport d'un tissu autologue vivant, faible cout et faible morbidité. La principale limite dans l'interprétation des résultats de ces séries reste l'hétérogénéité en termes de recul, taille et profondeur des lésions. Nous rapportons l'étude de Hangody *et al.* [2] qui représente la série la plus conséquente avec le recul le plus long qui comporte une série de 967 lésions chondrales avec 15 ans de recul. Le résultat était satisfaisant dans 92% des cas des lésions condyliennes, 87% des lésions tibiales et 74% des lésions fémoropatellaires.

**Tableau 2:** Présentation des principales séries ayant rapportées les résultats de la mosaïcoplastie

Série	Nombre des cas	Recul	Résultats satisfaisants
**Hangody et al [****2****]**	832	1 à 10 ans	92%
**Ollat et al [****3****]**	142	8 ans	81,8%
**Chow et al [****22****]**	36	3,8 ans	83%
**Jakob et al [****23****]**	52	3 ans	92%
**Marcacci et al [****29****]**	30	7 ans	77%
**Notre série**	35	2 ans	82,9%

Beaucoup d'éléments d'ordre général et local peuvent influencer le résultat final de la mosaïcoplastie. Ollat D *et al.* ont montré que les lésions de petites tailles et profondes, siégeant au niveau du condyle interne, opérées rapidement et chez un sujet de sexe masculin ont un pronostic meilleur. Ces constats ont été aussi prouvés dans notre étude en dehors du sexe masculin qui n'a pas été étudié. La taille du greffon est aussi importante, Robert a conclu dans son étude que les gros plots offrent une plus grande stabilité et une surface cartilagineuse plus importante au prix d'une morbidité élevée et d'un remplissage plus difficile (25). De même Ollat D *et al.* ont constaté dans leur étude étalée sur huit ans une tendance des chirurgiens à utiliser des plots plus gros, alors que Sgaglione *et al.* ont préconisé des petits plots de six à huit mm de largeur et de 10 à 15 mm de longueur [24]. Dans notre série qui s'intéresse seulement aux lésions condyliennes, nous avons utilisé des petits plots de trois à cinq mm de diamètre et nous avons trouvé 82% des résultats satisfaisants qui concordent parfaitement aux données de la littérature. Les autres alternatives thérapeutiques à savoir, les micro-fractures, malgré l'avantage d'un coup moindre et l'absence du prélèvement, donnent moins des résultats satisfaisants, se dégradant avec le temps. Cette constatation a été rapportée par une étude prospective randomisée réalisée par Gudas *et al.* [25] qui ont comparé 29 mosaïcoplasties contre 29 micro-fractures pour des lésions des 2,7 cm. Le résultat était largement supérieur pour la mosaïcoplastie avec 93% des résultats satisfaisants contre 49% pour les micro-fractures, ainsi qu'un meilleur résultat morphologique à l'IRM et une reprise plus rapide et plus performant des activités sportives. Pour les greffes des chondrocytes autologues de première génération, beaucoup des séries ont montré un résultat mois satisfaisant comparé à la mosaïcoplastie outre qu'elle nécessite deux interventions, deux anesthésies avec un temps de culture cellulaire à coup élevé. Deux équipes ont fait des études randomisées et comparatives avec un niveau de preuve considéré qui ont montré des résultats cliniques meilleurs et rapide avec un avantage sur le plan histologique pour la mosaïcoplastie [26,27]. Seul Bentley *et al.* ont trouvé un résultat contraire mais cette constatation ne peut pas être considérée vu que les lésions étaient volumineuses allant jusqu'au 12,2 cm et les greffons étaient de petits diamètres [28,29]. Pour les greffes chondrocytaires de deuxième et troisième génération, les techniques sont récentes et les études cliniques d'évaluation sont encore insuffisantes.

## Conclusion

Dans le domaine de la chirurgie cartilagineuse, l'absence des séries avec des reculs suffisants constitue la principale limite d'évaluation des résultats à long terme. Nul ne peut confirmer la supériorité d'une technique par rapport à l'autre mais tous les auteurs s'accordent à dire qu'il est nécessaire de corriger les causes directes et indirectes des lésions cartilagineuses à savoir le morphotype, la laxité et le capital méniscal. Il existe des zones entière d'incertitude ou des réponses insuffisantes comme les lésions tibiales, les lésions fémoropatellaires et les lésions en miroir [30,31]. Aucun consensus décisionnel n'a pu être proposé par les spécialistes de la chirurgie du cartilage mais nous pouvons, en analysant les données des séries et des conférences étudiées, proposer le démarche suivant :1) défect cartilagineux condylien inférieur à 2 cm^2^: c'est essentiellement les micro-fractures, mais la mosaïcoplastie peut être aussi indiquée de première intention. 2) Défect cartilagineux de 2 à 4 cm^2^: c'est la meilleure indication à a mosaïcoplastie. 3) Défect cartilagineux supérieur à 4 cm^2^: la greffe chondrocytaire semble être l'indication de choix compte tenu de la nature du tissu induit par les microfractures (fibrocartilage) et du sacrifice des zones donneuses pour les greffes ostéochondrales en mosaïque.

### État des connaissances actuelles sur le sujet

- L'ostéochondrite disséquante des condyles fémoraux est une affection fréquente;
- L'arsenal thérapeutique pour traiter cette affection est riche et les moyens thérapeutiques sont variés;
- La mosaîcoplastie est une alternative thérapeutique pour les pertes de substance cartilagineuse.

### Contribution de notre étude à la connaissance

- Résultats à moyen terme d'une série des malades traités par une mosaïcoplastie;
- Préciser les avantages et les inconvénients de cette mosaïcoplastie;
- Établir sa place dans l'arsenal thérapeutique ainsi que ses meilleures indications.

## Conflits des intérêts

Les auteurs ne déclarent aucun conflit d'intérêts.
